# Long-Term Effect of Adverse Childhood Experiences, School Disengagement, and Reasons for Leaving School on Delinquency in Adolescents Who Dropout

**DOI:** 10.3389/fpsyg.2020.02096

**Published:** 2020-12-14

**Authors:** Sung Man Bae

**Affiliations:** Department of Psychology and Psychotherapy, Dankook Univerisity, Cheonan, South Korea

**Keywords:** adverse childhood experiences, school disengagement, delinquency, school dropout, multivariate latent growth modeling

## Abstract

**Purpose**: The purpose of this study was to verify the long-term effect of adverse childhood experiences (ACEs), school disengagement, and the reasons for leaving school on adolescent delinquency while adjusting for sex.

**Methods**: Data were collected from 663 teenagers [male 368, female 295; mean age = 16.81 (*SD* = 1.10); age range = 13–19 years] through a Longitudinal Survey and Support Plan for Dropouts.

**Results**: Multivariate latent growth modeling (LGM) demonstrated that ACEs and school disengagement are positively associated with delinquency and the mediating effect of school disengagement on association between ACEs and delinquency was verified. Teenagers who quit school for personal reasons reported fewer instances of delinquent behavior than adolescents who left because of school reasons.

**Conclusion**: This study suggests that researchers and teachers need to consider ACEs and school disengagement as a warning index for delinquency with school dropout. In addition, the reason for leaving school should be considered to clarify the effect of school dropout on delinquency in adolescents who dropout.

## Introduction

In the Korean society, the rate of school dropout is estimated to be 0.6% in elementary school, 0.8% in middle school, and 1.6% in high school (Korean Educational Development Institute, 2018). Global estimates of school dropout rate vary by region: 4–12% in East Asia and Europe, and 43% in South and West Asia (Andrei et al., 2012; European Commission, 2013). Adolescents leave school for a variety of reasons (e.g., bullying, academic problems, and family economic difficulties) and youth who dropout may experience more daily stress (e.g., social prejudices). In particular, school dropout might be more likely to turn to delinquency than non-dropout might be (Bradshaw et al., 2008; Fernández-Suárez et al., 2016).

Various theoretical frameworks have explained the relationship between school dropout and delinquent behavior. General strain theory (GST) posits that daily stress leads to strain if strain persists, it can lead to delinquency. Based on this theory, school dropout increases strain and may consequently can cause delinquency (Agnew, 1992, 2002; Hoffmann, 2010; Nivette et al., 2017). The social bond theory argues that weakened ties with family and society can lead to delinquency. Based on the theory, school dropout weakens social ties and social control, and consequently increases delinquency (Özbay and Özcan, 2006; Hart and Mueller, 2013). Meanwhile, life-course perspective analyzes human behavior within structural, social, and cultural contexts. Based on the theory, school dropout might lead to delinquency, and the effect of school dropout on delinquency may vary depending on the reason and circumstances of dropping out (Sweeten et al., 2009; Na, 2017).

Empirical studies demonstrate inconsistent results on whether school dropout affects delinquency. Some studies indicate that school dropout increases the chance of delinquent behavior (Anderson, 2014; Fernández-Suárez et al., 2016), but other research argues that school dropout itself is not associated with delinquency (Drapela, 2004; Sweeten et al., 2009). Past literature implies that in order to prevent the delinquent behavior of students leaving school, finding a warning index of delinquency and antecedents of school dropout for juveniles is required.

Likewise, adverse childhood experiences (ACEs), including emotional abuse, physical neglect, and domestic violence such as parental divorce and having a family history of jail imprisonment (Rutter, 1983) are one of the key risk factors of delinquency (Mersky and Topitzes, 2010; Bellis et al., 2014; Baglivio et al., 2017). However, some studies assessed the presence of ACEs with single question, and many researchers studied the impact of ACEs on delinquency without considering its various types (Puszkiewicz and Stinson, 2019). In addition, the long-term effect of ACE on the delinquency behavior of students leaving school is not clear in the research that has been conducted thus far.

Similarly, school disengagement is associated with school dropout and delinquents (Henry et al., 2012; Snyder and Smith, 2015) and can be a warning index of delinquent behavior for school dropouts. School disengagement includes lateness, being absent, and not completing homework. Previous cross-sectional studies have argued that school disengagement is a major predictor of delinquent behavior (e.g., Henry and Thornberry, 2010). However, the long-term effect of school disengagement on delinquency for adolescents with school dropout is not clear.

ACE may affect school disengagement (Snyder and Smith, 2015). Similarly, a study by Bender (2012) verified the mediating effect of school engagement in the association between youth maltreatment and delinquency using latent growth curve modeling. However, further verification of the long-term mediating effect of school disengagement on the relationship between ACE and delinquency on school dropout appears to be necessary.

School dropout is not a sufficient factor that leads to delinquency. To clearly verify the long-term effect of school dropout on delinquency, researchers should explore other warning indices or antecedents of delinquency for teenagers who dropout of school. The reason for leaving school may also be a key predictor of delinquency. In other words, the effect of school dropout on delinquency may vary based on the reasons for dropping out (Sweeten et al., 2009). For example, leaving school for individual reasons such as general educational development (GED) is less likely to lead to delinquency than leaving school because of academic reasons (e.g., disliking school). Identity theory assumes that if the reason for leaving school is related to the formation of a positive identity (e.g., preparing the future career and family responsibility), the likelihood of delinquency decreases. In addition, the theory argues that leaving school because of academic reasons such as disliking study and academic failure increase the likelihood of delinquency (Markus and Nurius, 1986; Kiecolt, 1994).

Finally, sex can be a key covariate. The effect of sex on delinquent behavior is consistent in previous studies (Nivette et al., 2017; Cho and Lee, 2018; Hoffmann and Dufur, 2018).

The aim of this study is to examine the long-term effects of ACE, school disengagement, and the reasons for school dropout on delinquency while adjusting for sex. The hypotheses of this study are as follows:

*Hypothesis 1*: ACE would be positively related to delinquency in adolescents who dropout.
*Hypothesis 2*: School disengagement would be positively associated with delinquency in adolescents who dropout.
*Hypothesis 3*: Adolescents who leave school for personal reasons would engage in a lesser number of delinquent behaviors than students who leave for academic reasons.

## Materials and Methods

### Participants

This study used 1–3 waves of data from a Longitudinal Survey and Support Plan for Dropouts, which were obtained from the National Youth Policy Institute. In order to build a school dropout youth panel, individual lists provided by schools were utilized. The primary focus was on teenagers leaving middle school and high school. Panels were recruited through middle and high schools (349 adolescents), vocational training schools (57 adolescents), alternative educational institutions and GED institutes (167 adolescents), counseling centers (154 adolescents), and through web publicity (49 adolescents). In the first year (2013), 776 adolescents who had experienced school dropout participated in the survey. In the second year (2014), 599 adolescents (77.19% compared to the first year), and in the next year (2015), 549 adolescents (70.75% compared to the first year) participated in the survey. Data from 663 youths [male = 368, female = 295; mean age = 16.81 (*SD* = 1.10); age range = 13–19 years] were used for the final analysis, excluding 113 participants with a high non-response rate. The survey was conducted using face-to-face interviews. In case of institutional contact, face-to-face interviews and self-reported group surveys were conducted. This study used data freely available to the public and hence did not require ethical approval. Tables 1 and 2 describe the specific socio-demographic characteristics of participants and descriptive statistics of the variables in this study.

**Table 1:** Demographic characteristics of participants.

Variable		*N*	%
Gender	Male	368	55.5
	Female	295	44.5
Age	13	6	0.9
	14	21	3.2
	15	41	6.2
	16	157	23.7
	17	254	38.3
	18	171	25.8
	19	13	2.0
Reasons of dropout	Individual reasons	253	38.2
	Academic reasons	410	61.8
Current state after dropout	Dropout	625	94.3
	Returned	38	5.7

*N* = 663.

**Table 2:** Descriptive statistics of the variables.

	Min	Max	Mean	*SD*	*N*
Delinquency 1 (2013)	0	8	1.73	1.63	663
Delinquency 2 (2014)	0	8	1.79	1.52	559
Delinquency 3 (2015)	0	8	1.77	1.36	432
ACE	0	12	2.77	2.48	663
School disengagement	0	16	6.38	4.64	663

*N* = 663; ACE, adversity childhood experiences.

### Measures

#### Adverse Childhood Experiences

The scale developed by Lee et al. (2005) consists of 19-items that assess the presence of parental death, parental divorce, parental remarriage, parents who leave the family, parental business failure, parental unemployment, parental illness, bitter quarrels between parents, physical abuse, and abuse by teachers. Each item is coded as a dichotomous variable (absent = 0, present = 1). Higher total scores indicate greater childhood adversity.

#### School Disengagement

The questionnaire developed by National Youth Policy Institute (2013) consists of four items with a five-point scale (never = 0, almost every day = 4) that measure lateness, not doing homework, being absent from class, and a confrontation with the teacher. Higher total scores are related to higher degrees of school disengagement. Cronbach’s alpha for this scale was 0.843 for the current sample.

#### Reasons for School Dropout

Reasons for leaving school were coded as two categories (Sweeten et al., 2009). Individual reasons included family economic difficulties, health problems, career preparing, and the GED. Academic reasons included school maladjustment, poor grades, and a dislike for teachers/peers/studying/school.

#### Delinquency

The questionnaire developed by Choi et al. (2011) was used to assess delinquent behavior, which consists of eight items and measures the presence of smoking, alcohol use, stealing, extortion of money, viewing obscene material, gambling for money, vandalism, and violence toward others. Each item is coded as a dichotomous variable (absent = 0, present = 1). Higher total scores indicate higher number of delinquent behaviors.

#### Covariates

Sex (male = 0 and female = 1) was set as the covariate.

### Analysis

**Figure 1:**
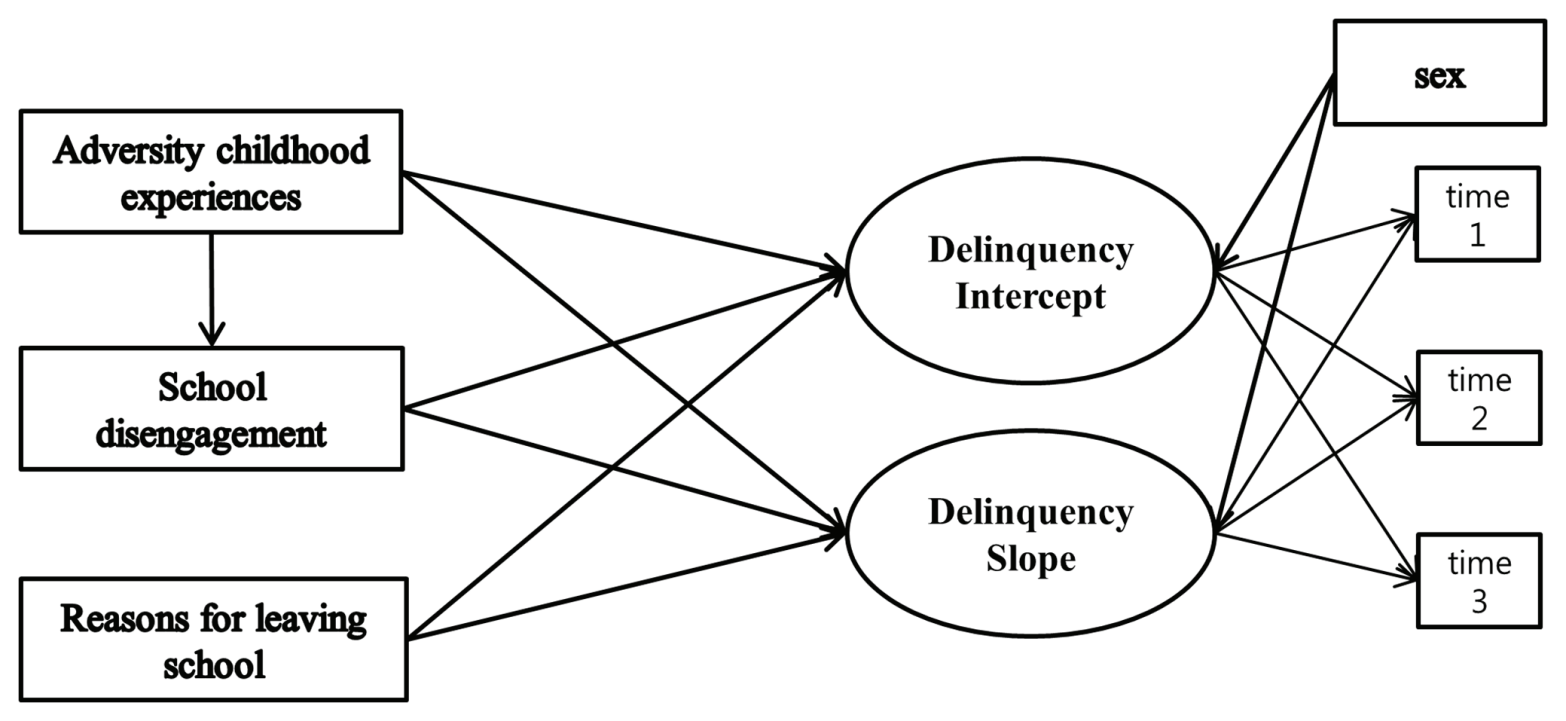
Multivariate latent growth model.

The research model (Figure 1) was examined using latent growth modeling (LGM) by the AMOS 20.0 program. LGM is a useful method to verify the long-term association between changes in the parameters of variables. In first step, univariate LGM was conducted in order to examine the changing trend in delinquency. In this study, the linear growth model and no growth model were compared based on the average trend of delinquency. In the second step, multivariate LGM was performed to test the long-term effect of ACE, school disengagement, and reasons for school dropout on delinquency. Specifically, the paths from each independent variable to the intercept and slope of delinquency were set. Intercept means the average value of delinquency at time 1, and slope indicates the rate of change of delinquency over time. The fitness of the research model was tested through fit indices, such as the chi-square value, comparative fit index (CFI), the Tucker-Lewis index (TLI), and root mean square error of approximation indices (RMSEA). TLI and CFI are regarded as acceptable if the index is higher than 0.95, and RMSEA is acceptable if the index is lower than 0.05. A bootstrapping test was performed to test the indirect effects of school disengagement on the association between ACE and delinquency.

## Results

### Univariate LGM

A univariate LGM was used to test the changing trend of delinquency. The fit of the linear growth model of delinquency (chi-square value = 0.123, *df* = 1, TLI = 1.006, CFI = 1.000, RMSEA = 0.000) was better than the fit of the no growth model (chi-square value = 29.877, *df* = 2, TLI = 0.910, CFI = 0.940, RMSEA = 0.145), which means that delinquency increased linearly over time.

### Multivariate LGM

#### Fitness of the Research Model

The chi-square value of the research model was 10.387 (*p* > 0.05). The TLI and CFI were 0.987 and 0.996, respectively, and the RMSEA was 0.027. The fitness of the research model is acceptable (Figure 1).

#### Direct Effect Between Variables

The direct effects are indicated in Table 3. First, the intercept of ACE had a positive effect on the intercept of delinquency (*β* = 0.138, *t* = 6.761, *p* < 0.001). The intercept of ACE had a negative impact on the change rate of delinquency (*β* = −0.049, *t* = −4.426, *p* < 0.001). The results indicated that higher ACE scores were related to an increase in frequency of delinquent behavior, and the effect of ACE scores on the increasing rate of delinquency decreased over time.

**Table 3:** Path coefficients of multivariate latent growth modeling (LGM).

Path	*β*	*B*	S.E.	C.R.
ACE intercept → D intercept	0.138	0.268	0.020	6.761^***^
ACE intercept → D slope	−0.049	−0.252	0.011	−4.426^***^
SD intercept → D intercept	0.125	0.454	0.011	10.992^***^
SD intercept → D slope	−0.038	−0.372	0.006	−6.270^***^
RFLS → D intercept	0.337	0.128	0.107	3.139^**^
RFLS → D slope	−0.111	−0.113	0.058	−1.922^+^
ACE → SD	0.207	0.111	0.070	2.985^**^
Sex → D intercept	−0.840	−0.327	0.101	−8.282^***^
Sex → D slope	0.122	0.127	0.055	2.237^*^

* *p* < 0.05;

+ *p* < 0.06;

** *p* < 0.01;

*** *p* < 0.001.

ACE, adversity childhood experiences; SD, school disengagement; D, delinquency; RFLS, reasons for leaving school.

Second, the intercept of school disengagement had a positive effect on the intercept of delinquency (*β* = 0.125, *t* = 10.992, *p* < 0.001). The intercept of school disengagement had a negative effect on the change rate of delinquency (*β* = −0.038, *t* = −6.270, *p* < 0.001). The results indicate that increased school disengagement is related to an increased frequency of delinquent behavior, and the effect of school disengagement on the increasing rate of delinquency decreased over time.

Third, the intercept of the reason for school dropout had a positive effect on the intercept of delinquency (*β* = 0.337, *t* = 3.139, *p* < 0.01). The intercept of the reason for school dropout had a negative influence on the change rate of delinquency (*β* = −0.111, *t* = −1.922, *p* < 0.06). The results demonstrated that adolescents leaving school for individual reasons reported fewer instances of delinquent behaviors than teenagers who experience school dropout because of academic reasons.

The intercept of sex had a negative effect on the intercept of delinquency (*β* = −0.840, *t* = −8.282, *p* < 0.001). However, the intercept of sex had a positive impact on the change rate of delinquency (*β* = 0.122, *t* = 2.237, *p* < 0.05). The results indicated that male students reported more instances of delinquent behaviors than female students and the increase in rate of delinquency in male students increased over time.

#### Indirect Effect of School Disengagement

A bias-corrected bootstrap test was conducted to examine the mediating effect of school disengagement on the relationship between ACE and delinquency. The indirect effect of the initial value of ACE had a positive impact on the initial value of delinquency through the initial value of school disengagement (*β* = 0.026, *p* < 0.05; CI, LO = 0.008, UP = 0.046). Also, the direct effect of the initial value of ACE influenced the initial value of delinquency (*β* = 0.138, *t* = 6.761, *p* < 0.001). The results indicated that the initial value of school disengagement had a partial mediating effect on the relationship between initial value of ACE and the initial value of delinquency.

## Discussion

The purpose of this study was to examine the long-term effects of ACE, school disengagement, and reasons for leaving school on delinquency in students who dropout while adjusting for sex. The implications and discussions for the major results are as follows.

ACE is positively related to delinquency, and the long-term effect of ACE on delinquency has been identified in this study. Exposure to adverse events in childhood does not necessarily lead to delinquency. However, ACE may promote distrust, hostility, and a cynical attitude toward social norms and may consequently lead to delinquency (Kabiru et al., 2014). According to the GST, an increasing number of life stress events lead to strain, which in turn, increase the chance of delinquency (Hoffmann, 2010). In particular, this study assessed various types of childhood adversity and verified the long-term effect of ACE on delinquency in teenagers with school dropout. The results imply that counselors and researchers should understand the student’s ACE severity to prevent delinquent behavior in adolescents who experience school dropout.

School disengagement was positively associated with delinquency, and the long-term effect of school disengagement has been verified. A number of researchers have argued that the effect of school dropout on delinquency is weak, and the impact of other antecedent variables such as school engagement is more important than dropout (Drapela, 2004; Henry et al., 2012). According to social bond theory, school disengagement is related to lack of social ties and social control, which may increase the chance of delinquency. The results indicate that school disengagement can be a warning index of delinquent behavior for students with school dropout. In addition, the mediating effect of school disengagement in the association between ACE and delinquency suggests that researchers and counselors should pay attention to the school disengagement of students with high ACE scores to prevent delinquency with school dropout.

An important result of this study is the link between the reasons for dropping out and delinquency. A few studies have examined the effect of the reasons for leaving school on delinquency (Laub and Sampson, 2003; Na, 2017). According to the results of this study, it is estimated that students who leave school for personal reasons (e.g., general education development test) are unlikely to become delinquents when compared to students who leave school for academic reasons. They have a new positive social identity after leaving school, which reduces strain during school life and extends positive social networks (Sweeten et al., 2009), and consequently helps to prevent delinquency.

The main contribution of this study is that it has identified that ACE and school disengagement can be key warning indices of delinquency in teenagers who dropout of school. This study also suggests that the reason for leaving school may be a more important variable than school dropout itself to predict delinquency.

The limitations of this study and suggestions for future research are as follows. School disengagement was measured by means of a self-report. However, for more accurate measurements, it is necessary to use official information (e.g., the number of absent days from official school records). In future studies, in order to understand more accurately the effect of school dropout on delinquency, it is necessary to verify the relationship between the antecedent variables or warning indices, school dropout, and delinquency. Finally, the findings are limited by the content of the existing dataset used for the analysis. The findings might have changed with the additional other variables, which were not available in the database at the time of this study.

## Data Availability Statement

Publicly available datasets were analyzed in this study. This data can be found here: https://archive.nypi.re.kr/brdartcl/boardarticleList.do?brd_id=BDIDX_ey87ybjme4Hx3Tjqb76kA9&menu_nix=65N4O6xd.

## Ethics Statement

Ethical approval was not provided for this study on human participants because publicly available datasets were analyzed in this study. Written informed consent to participate in this study was provided by the participants’ legal guardian/next of kin.

## Author Contributions

SB performed study design, statistical analysis, and writing.

### Conflict of Interest

The author declares that the research was conducted in the absence of any commercial or financial relationships that could be construed as a potential conflict of interest.

## Abbreviations

ACE: Adverse childhood experiences
LGM: Latent growth modeling.
